# Critical Endothelial Regulation by LRP5 during Retinal Vascular Development

**DOI:** 10.1371/journal.pone.0152833

**Published:** 2016-03-31

**Authors:** Wei Huang, Qing Li, Mahmood Amiry-Moghaddam, Madoka Hokama, Sylvia H. Sardi, Masashi Nagao, Matthew L. Warman, Bjorn R. Olsen

**Affiliations:** 1 Department of Developmental Biology, Harvard School of Dental Medicine, and Department of Cell Biology, Harvard Medical School, Boston, Massachusetts, United States of America; 2 Translational Science Lab, Diagnostic Imaging and Biomedical Technology, GE Global Research Center, Niskayuna, New York, United States of America; 3 Institute of Basic Medical Sciences, University of Oslo, Oslo, Norway; 4 Okayama University Medical School, Okayama, Japan; 5 Howard Hughes Medical Institute and Orthopaedics Research Laboratories, Boston Children’s Hospital, and Departments of Genetics and Orthopaedic Surgery, Harvard Medical School, Boston, Massachusetts, United States of America; Katholieke Universiteit Leuven, BELGIUM

## Abstract

Vascular abnormalities in the eye are the leading cause of many forms of inherited and acquired human blindness. Loss-of-function mutations in the Wnt-binding co-receptor LRP5 leads to aberrant ocular vascularization and loss of vision in genetic disorders such as osteoporosis-pseudoglioma syndrome. The canonical Wnt-β-catenin pathway is known to regulate retinal vascular development. However, it is unclear what precise role LPR5 plays in this process. Here, we show that loss of LRP5 function in mice causes retinal hypovascularization during development as well as retinal neovascularization in adulthood with disorganized and leaky vessels. Using a highly specific *Flk1-Cre*^*Breier*^ line for vascular endothelial cells, together with several genetic models, we demonstrate that loss of endothelium-derived LRP5 recapitulates the retinal vascular defects in *Lrp5*^*-/-*^ mice. In addition, restoring LRP5 function only in endothelial cells in *Lrp5*^*-/-*^ mice rescues their retinal vascular abnormalities. Furthermore, we show that retinal vascularization is regulated by LRP5 in a dosage dependent manner and does not depend on LRP6. Our study provides the first direct evidence that endothelium-derived LRP5 is both necessary and sufficient to mediate its critical role in the development and maintenance of retinal vasculature.

## Introduction

Vision impairment and blindness are devastating conditions afflicting over 4% of the world population [1]. In developed countries, vascular abnormalities are the major cause of many forms of inherited and acquired human blindness, such as Osteoporosis-Pseudoglioma Syndrome (OPPG), Norrie Disease (ND), Familial Exudative Vitreoretinopathy (FEVR) and diabetic retinopathy (DR) [2,3]. Both aberrant vascular development and pathological neovascularization can critically impair the high metabolic activities in the retina. The retinal vasculature consists of three vessel beds located in the nerve fiber layer (NFL), inner plexiform layer (IPL) and outer plexiform layer (OPL). Its heavy reliance on a well-timed and balanced orchestration of many factors involving different cell types, multiple signaling inputs and proper oxygen levels makes it susceptible to anomalies that are difficult to study [4]. However, some of these blinding conditions have overlapping genetic causes and/or ocular manifestations, indicating that they likely have shared pathological mechanisms. Therefore, studies of human genetic ocular disorders have provided insights into biological and pathological processes that also underlie acquired diseases. Here, in the context of OPPG, we present data on the critical role of low-density lipoprotein receptor-related protein-5 (LRP5) during retinal vascular development.

OPPG is a rare autosomal recessive disorder characterized by severe childhood osteopenia and congenital or infancy-onset visual loss [5–7]. Major manifestations in the eye include retinal hypovascularization, retrolental fibrovascular tissue (pseudoglioma), microphthalmia and various vitreoretinal abnormalities. The disorder is caused by loss-of-function mutations in LRP5, a co-receptor in the canonical Wnt signaling pathway. Many of the ocular findings in OPPG patients overlap with those of FEVR and ND, caused by loss-of-function mutations in other Wnt signaling components, such as Frizzled-4 (FZD4) and Norrie disease protein (NDP) [8–12].

Seminal studies by the Nathans group and others have shown that Müller glial cells secrete Norrin that binds to FZD4 in endothelial cells (ECs) and regulates retinal vascular development through the canonical Wnt-β-catenin pathway [13–16]. Disruption of this pathway through loss of Norrin, FZD4 or LRP5 function not only leads to an overlapping spectrum of ocular problems in patients, but also results in similar retinal vascular defects in mice. Mice in which *Fzd4* is conditionally knocked out (CKO mice) by using *Tie2‐Cre* (*Tie2‐Cre;Fzd4*^*fl/-*^) exhibit the retinal hypovascularization phenotype seen in *Fzd4* null (*Fzd4*^*-/-*^) and *Ndp* null (*Ndp*^*-*^) mice, and inducing β-catenin function in ECs rescues these defects in *Ndp*^*-*^ mice [14,17]. Based on these data, it has been proposed that the pathway functions in ECs to control retinal vascularization. However, cells that express *Tie2-Cre* include not only ECs but also several other cell types [18], indicating a possible contribution of non-EC-derived FZD4 to retinal vascular regulation. Furthermore, inducing β-catenin activity in ECs may bypass the need for Norrin-FZD4-β-catenin signaling in non-ECs. In addition, although activation of the Norrin-FZD4-β-catenin pathway requires the presence of either LRP5 or LRP6 *in vitro* [14], it is unclear what exact roles LRP5 and LRP6 play during retinal vascular development *in vivo*, and what cell types contribute to the incomplete vascularization in the retina of OPPG patients and *Lrp5*^*-/-*^ mice.

In this study, we use multiple genetic animal models to address these questions. Our use of a highly endothelial-specific *VEGF receptor 2-Cre* line (*Flk1-Cre*^*Breier*^) makes it possible, for the first time, to evaluate the critical function of LRP5 in ECs during retinal vascular development.

## Results

### An Essential Role of LRP5 in Retinal Vascular Development

**Fig 1:**
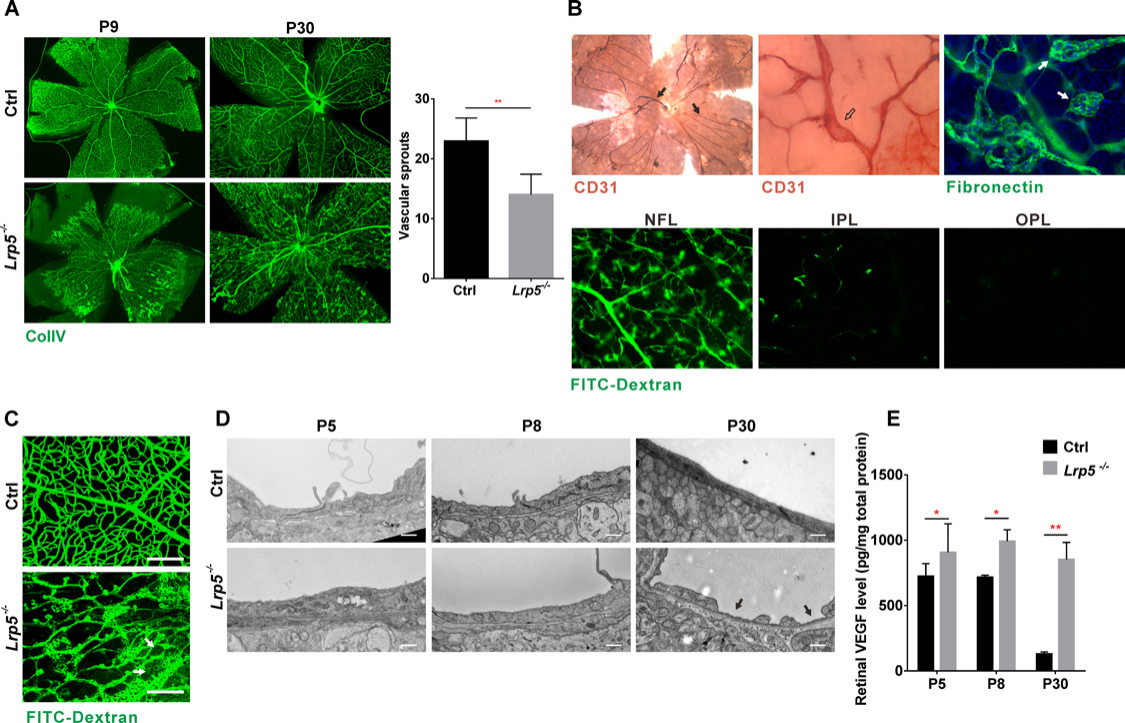
Loss of *Lrp5* causes retinal hypovascularization and neovascularization. (A) ColIV whole mount IF staining showing retinal vessels of *Lrp5*^*-/-*^ and control mice at P9 and P30. Quantification of vascular sprout numbers at P5 shown at right. (B) Adult *Lrp5*^*-/-*^ retinas showing persistent hyaloid vessels (black arrows, CD31 IHC staining), aneurysms (open arrow, CD31 IHC staining), neovascular overgrowth (white arrows, fibronectin IF staining) and lack of IPL and OPL vascular development (lower panels, green: FITC-Dextran perfusion). (C) FITC-Dextran perfusion (green) showing retinal vascular leakage (white arrows) in adult *Lrp5*^*-/-*^ mice compared to control. Scale bars = 100μm. (D) EM analysis of endothelium of *Lrp5*^*-/-*^ and control retinas at P5, P8 and P30. Arrows point to area of endothelial fenestration. Scale bars = 500nm. (E) Total amounts of VEGF protein in retinas of *Lrp5*^*-/-*^ and control mice at P5, P8 and P30. Each ELISA was done in duplicate and normalized to total retinal protein amount. *n* = 9, 5, 12 for controls (at P5, P8, P30) and 9, 8, 8 for *Lrp5*^*-/-*^ (at P5, P8, P30). * *p*<0.05, ** *p*<0.01. Data are represented as means ± SD. Ctrl, control.

To examine the role of LRP5 in retinal vascular development, we first analyzed the retinal vasculature in *Lrp5*^-/-^ mice at different developmental stages in detail. Retinal whole mount immunofluorescence (IF) staining of collagen IV (ColIV) showed that *Lrp5*^-/-^ retinas exhibited retarded endothelial outgrowth with sparse vessel coverage in the NFL during early postnatal development (Fig 1A). In adult *Lrp5*^-/-^ mice, retinal angiography with FITC-dextran perfusion showed that the intraretinal vessel layers in the IPL and OPL were mostly absent, with occasional incomplete vascular development in the IPL, and vessels penetrating from the NFL terminated in clusters without branching (Fig 1B and S1 Movie and S2 Movie). Intravitreal hemorrhage was frequently observed and hyaloid vessels persisted into adulthood, long after the time when they normally regressed in WT mice (Fig 1B). These retinal hypovascularization defects in *Lrp5*^-/-^ mice confirm findings in previous studies [14,19]. In addition, the NFL exhibited chaotic vessel overgrowth (neovascularization) with arterio-venous anastomoses, microaneurysms, convoluted neovascular tufts, and leaky vessels (Fig 1B and 1C). Electron microscopy (EM) showed widespread endothelial fenestrations (EF) in adult *Lrp5*^-/-^ retinas but not during early postnatal development (Fig 1D), indicating that EF is unlikely a primary defect caused by loss of *Lrp5*. As increased levels of VEGF can often lead to endothelial fenestration [20], we examined retinal VEGF amounts in the mice through ELISA assays. As shown in Fig 1E, total retinal VEGF levels in *Lrp5*^-/-^ mice were only slightly increased, about 1.3-fold, at P5 and P8 compared to controls, but were 6.6-fold higher in adults. This suggests that elevated retinal VEGF is likely a secondary response to the retinal hypovascularization defect in *Lrp5*^-/-^ mice during postnatal development and a contributor to EF defects when it reaches a high level in adulthood.

### LRP5 Signaling in *Tie2*^+^ Cells but Not *VE-Cadherin*^+^ Cells is Essential for Retinal Vascular Development

**Fig 2:**
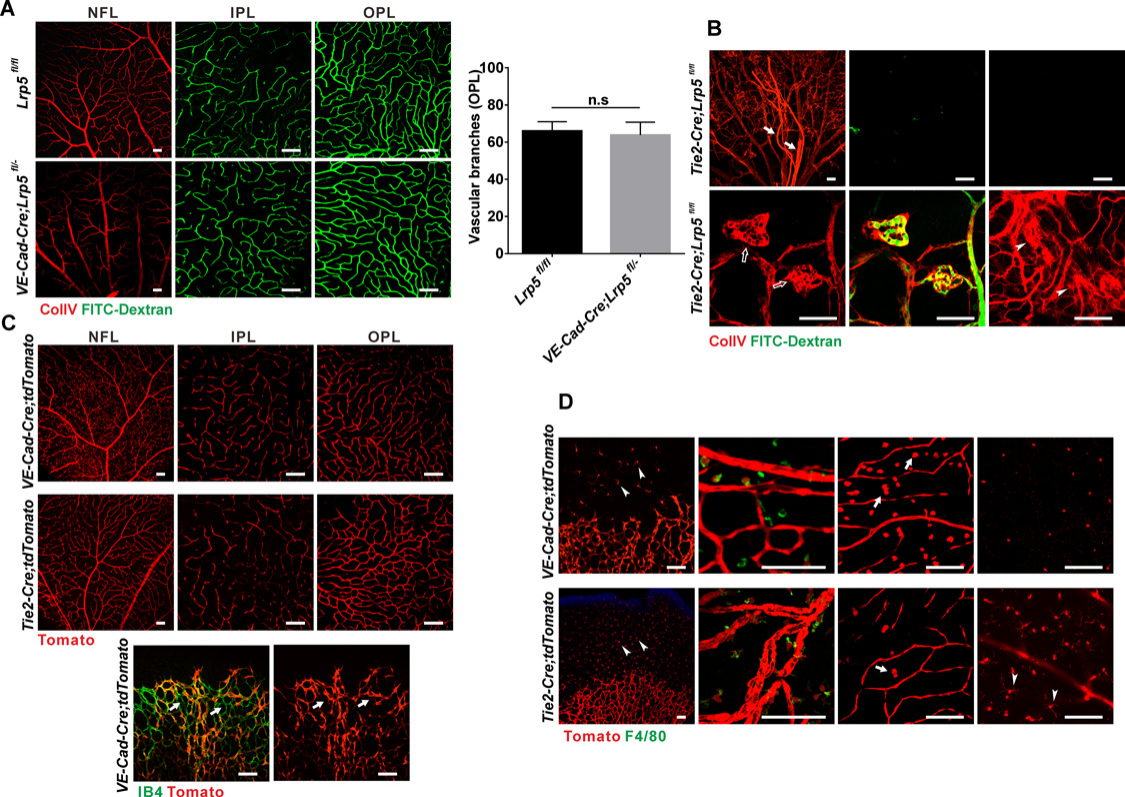
Conditional knockout of *Lrp5* with *Tie2-Cre* but not *VE-Cad-Cre* recapitulates retinal vascular defects in *Lrp5*^*-/-*^ mice. (A and B) ColIV IF staining (red) and FITC-Dextran perfusion (green) showing adult retinal vasculature in *VE-Cad-Cre;Lrp5*^*fl/-*^ and *Tie2-Cre;Lrp5*^*fl/fl*^ CKO mice (4w) compared to *Lrp5*^*fl/fl*^ control. Arrows: hyaloid vessels; open arrows: neovascular tufts; arrowheads: neovascular overgrowth. At right: Quantification of vascular branch points in OPL at 4w; ns not significant. (C) TdTomato signals in *VE-Cad-Cre;tdTomato* (8w) and *Tie2-Cre;tdTomato* (7w) mice showing predominant endothelial expression of *VE-Cad-Cre and Tie2-Cre* in the NFL, IPL and OPL of the retina. Note that *VE-Cad-Cre* is also widely expressed in myeloid cells around the vessels in the NFL (upper left panel, also see D). Arrows point to IB4 (green) positive vascular area with negative *VE-Cad-Cre;tdTomato* signals in a P5 mouse indicating incomplete *VE-Cad-Cre* recombination. (D) Myeloid and microglial localization of *VE-Cad-Cre;tdTomato* and *Tie2-Cre;tdTomato* signals in P5 (left and mid-left panels) and adult (right and mid-right panels) retinas. Arrows point to myeloid cells and arrowheads point to microglial cell. Green: F4/80 IF staining. Scale bars = 100nm.

In the retina, *Lrp5* is expressed predominantly in Müller glia and in ECs [19,21]. To identify the primary cell population requiring *Lrp5* expression for retinal vascularization, we used mice with *Lrp5* floxed alleles [22] to conditionally knock out *Lrp5* in retinal neural/glial cells using *Rx-Cre* [23] and in ECs using *VE-cadherin-Cre* (*VE-Cad-Cre*) [24] or *Tie2-Cre* mice [25]. Loss of *Lrp5* in retinal neural/glial cells had no impact on retinal vessels (S3 Movie). Surprisingly, while *VE-Cad-Cre;Lrp5*^*fl/-*^ CKO mice (harboring one floxed and one null *Lrp5* allele) also demonstrated a completely normal retinal vasculature (Fig 2A), *Tie2-Cre;Lrp5*^*fl/fl*^ mice exhibited vascular defects that were almost identical to those of *Lrp5*^*-/-*^ mice. Briefly, the hyaloid vessels failed to regress, intraretinal vascular beds were absent in adult mice, vessels penetrating from the NFL terminated in club-like clusters and the NFL exhibited chaotic neovascularization (Fig 2B).

As both *VE-Cad-Cre* and *Tie2-Cre* transgenes are predominantly expressed in ECs [24,25] and are widely used in EC-related genetic studies, this large discrepancy of phenotypes in *VE-Cad-Cre;Lrp5*^*fl/-*^ and *Tie2-Cre;Lrp5*^*fl/fl*^ CKO mice casts doubt on the conclusion that EC-dependent LRP5 signaling is critical for retinal vascular development. First, *VE-Cad-Cre*^+^ and *Tie2-Cre*^+^ cells are known to give rise to half and most adult hematopoietic cells, respectively, in addition to ECs [18,24,26]. Therefore, we considered the possibility that the essential function of LRP5 may be associated with *Tie2-Cre*^+^ hematopoietic cells rather than the *Tie2-Cre*^+^ ECs. Secondly, although we increased the *Lrp5* knockout efficiency in *VE-Cad-Cre;Lrp5*^*fl/-*^ CKO mice by incorporating one *Lrp5* null allele, we also considered the possibility that *VE-Cad-Cre* may have a much lower recombination efficiency in retinal ECs than *Tie2-Cre*, and that incomplete endothelial deletion of *Lrp5* in *VE-Cad-Cre;Lrp5*^*fl/-*^ mice may explain the lack of a vascular defect. To distinguish between these possibilities, we further investigated the expression of *VE-Cad-Cre* and *Tie2-Cre* in the eye.

### Endothelial and Non-endothelial Expression of *VE-Cadherin-Cre* and *Tie2-Cre* in the Retina

Using a tdTomato reporter line [27], we generated *VE-Cad-Cre*;*tdTomato* and *Tie2-Cre*;*tdTomato* double transgenic mice. Whole-mount retinal microscopy showed that both *VE-Cad-Cre* and *Tie2-Cre* were predominantly expressed in retinal ECs in all three vascular beds (Fig 2C). However, vascular patches with negative *VE-Cad-Cre*;*tdTomato* signals could often be observed in the developing retina (Fig 2C), indicating incomplete recombination with *VE-Cad-Cre* in endothelium. *Tie2-Cre*^+^ and *VE-Cad-Cre*^+^ signals were also widely present in microglia and macrophage/myeloid cells (Fig 2D), but their distribution in these cells differed developmentally.

In the first postnatal week, compared to *VE-Cad-Cre*, the *Tie2-Cre;tdTomato* signal was seen in much more microglial cells both ahead of the vascular leading edge and around the developing vessels (Fig 2D). Whole mount IF staining with F4/80, a macrophage marker, showed that almost all F4/80^+^ macrophage/myeloid cells in the NFL had a *Tie2-Cre*^+^ origin while only about half were *VE-Cad-Cre;tdTomato*^+^ (Fig 2D). In adult mice, a very large number of myeloid cells around the NFL vessels showed *VE-Cad-Cre;tdTomato* signals with only a few showing signs of being derived from *Tie2*^+^ cells (Fig 2D). However, intraretinal microglial cells still presented strong tdTomato signals from a *Tie2*^+^ but not *VE-Cad*^+^ origin (Fig 2D).

These data indicate that compared to *Tie2-Cre*, *VE-Cad-Cre* is less efficient in recombining targets in retinal ECs, and this may explain the lack of any vascular defects in *VE-Cad-Cre;Lrp5*^*fl/-*^ CKO mice. At the same time, *Tie2*^+^ cells also give rise to myeloid and microglial cells in the retina, thus not excluding a possible contribution of these cells to the vascular phenotype in *Tie2-Cre;Lrp5*^*fl/fl*^ CKO mice.

### LRP5 Signaling in Endothelial but Not Myeloid Cells is Essential for Retinal Vascular Development

**Fig 3:**
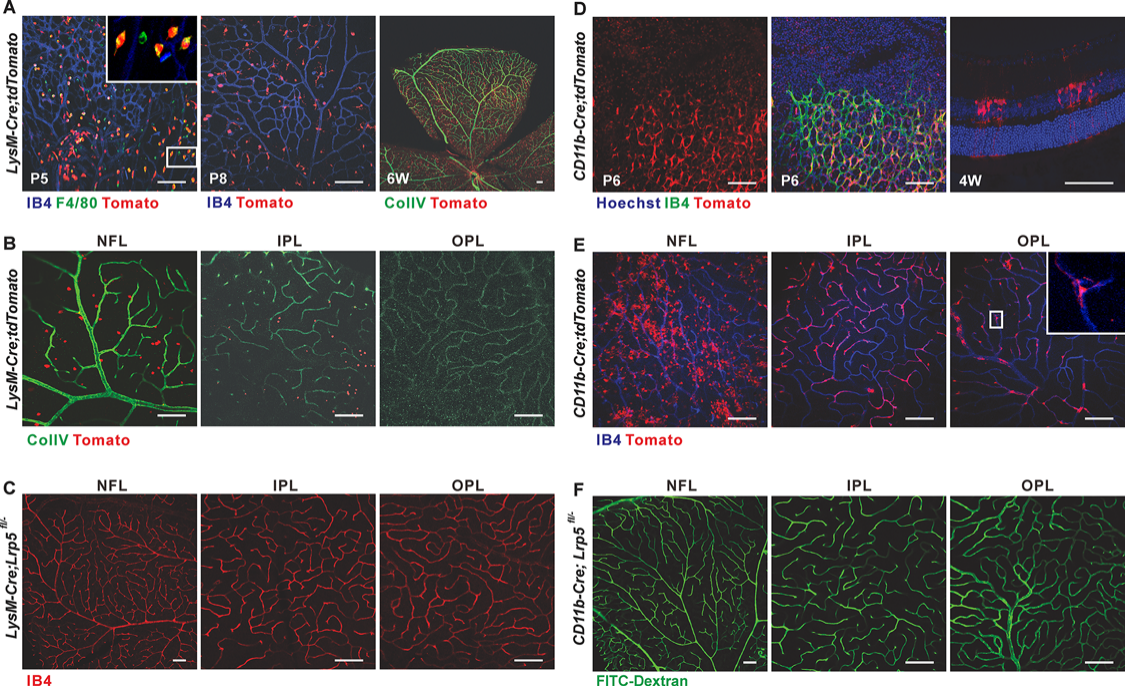
Retinal vascular phenotype in mice with conditional knockout of *Lrp5* in myeloid/microglial cells. (A) Whole mount IF staining with IB4 (blue) or ColIV antibody (right panel, green) showing distribution of *LysM-Cre;tdTomato*^+^ (red) myeloid and microglial cells in retinas at P5, P8 and 6w. Most *LysM-Cre;tdTomato*^+^ myeloid cells were also F4/80^+^ (green) (white rectangle in left panel indicates area shown at higher magnification in the upper right corner inset). (B) Localization of *LysM-Cre;tdTomato*^+^ myeloid cells in three adult (6w) retinal vascular beds. Green: ColIV. (C) Retinal vasculature of *LysM-Cre;Lrp5*^*fl/-*^ CKO mice (12w). Red: IB4. (D) Distribution of *CD11b-Cre;tdTomato*^+^ (red) myeloid and microglial cells in retinas at P6 (left and mid panels, green: IB4) and 4w (right panel, cross section). Blue: Hoechst. (E) Localization of *CD11b-Cre;tdTomato*^+^ signals (red) in myeloid, microglial, Müller glial and perivascular macrophage cells in adult (4w) retina. Blue: IB4. White rectangle in right panel outlines cell shown at higher magnification in upper right corner inset. (F) Retinal vasculature of *CD11b-Cre;Lrp5*^*fl/-*^ CKO mice (4w). Green: FITC-Dextran perfusion. Scale bars = 100nm.

To further examine the potential contributions of *Tie2*^+^ myeloid/microglial cells to the vascular phenotype in *Tie2-Cre;Lrp5*^*fl/fl*^ CKO mice, we used two *in vivo* strategies. First, we conditionally deleted *Lrp5* in myeloid/glial cells with M lysozyme-*Cre* (*LysM-Cre*) [28] and *CD11b-Cre* [29]. Both *LysM-Cre* and *CD11b-Cre* have been shown to have a high degree of recombination in bone marrow macrophages and granulocytes as well as peritoneal macrophages [28,29]. While *CD11b-Cre* also demonstrates recombination activity in brain microglial cells and *LysM-Cre* is less effective in tissue macrophages, the expression of these *Cre’s* in the retina is unclear. We generated double transgenic mice of tdTomato with these two *Cre* lines and showed that *LysM-Cre* was primarily expressed in myeloid and microglial cells in developing retinas and mostly in myeloid cells around adult NFL (Fig 3A and 3B). *CD11b-Cre* was abundantly expressed in microglia and Müller glia both in developing and mature retinas (Fig 3D and 3E). Nonetheless, both *LysM-Cre;Lrp5*^*fl/-*^ and *CD11b-Cre;Lrp5*^*fl/-*^ CKO mice developed a three-tier retinal vasculature similar to controls (Fig 3C and 3F), indicating that myeloid/microglial LRP5 is dispensable for retinal vascularization.

**Fig 4:**
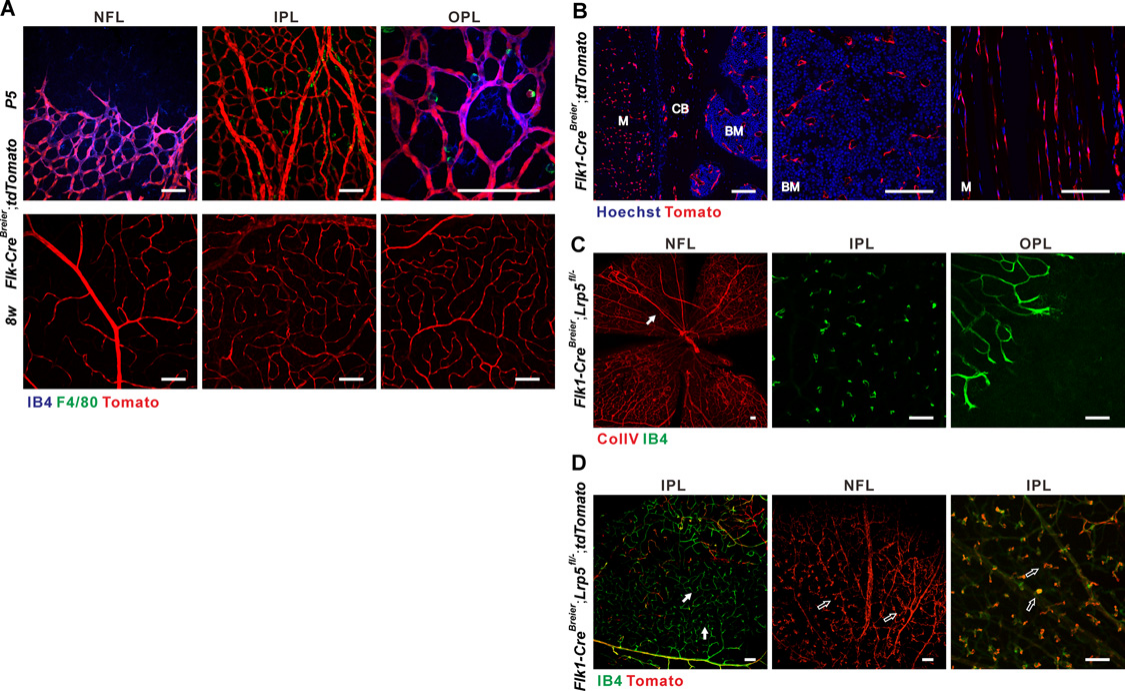
*Flk1-Cre*^*Breier*^ is specifically expressed in endothelial cells and conditional knockout of *Lrp5* with *Flk1-Cre*^*Breier*^ recapitulates retinal vascular defects in *Lrp5*^*-/-*^ mice. (A) Specific endothelial location of *Flk1-Cre*^*Breier*^*;tdTomato* signals in developing retinas (P5, upper panels) overlapping with IB4 IF signals (blue). F4/80 IF staining (green) showing that most macrophages were tdTomato negative. In adult retina, *Flk1-Cre*^*Breier*^ was specifically expressed in all three layers of the retinal vascular beds (lower panels) with no signs of any myeloid cell expression. Penetrance of *Flk1-Cre*^*Breier*^ expression in ECs could also be incomplete as shown in (D). (B) Specific endothelial location of *Flk1-Cre*^*Breier*^*;tdTomato* signals in adult (4w) bone marrow, cortical bone and skeletal muscle. M: muscle; CB: cortical bone; BM: bone marrow. Blue: Hoechst. (C) Whole mount IF staining of ColIV (red) and IB4 (green) showing retinal vasculature in adult (4w) *Flk1-Cre*^*Breier*^*;Lrp5*^*fl/-*^ CKO mice with disorganized NFL vessels, vertical vessel branches terminating in ball-like structures in the IPL and lack of OPL vascular bed. Arrow points to persistent hyaloid vessels. Note that regions with vascular abnormalities often had patchy normal-looking areas located in the neighborhood. In the selected OPL image, an area with well-developed vessels is next to another with no vascular development. (D) Whole mount IF staining of IB4 (green) showing that normally developed vascular areas in *Flk1-Cre;Lrp5*^*fl/-*^*;tdTomato* retinas (8w) included many *Flk1-Cre;*tdTomato negative vessels (arrows), whereas abnormal vessel structures were all tdTomato positive (open arrows). Scale bars = 100μm.

Next, we generated another endothelial *Lrp5* CKO with VEGF receptor 2-driven *Cre* (*Flk1-Cre*^*Breier*^) transgenic mice [30]. Unlike *Tie2-Cre* and *VE-Cad-Cre*, *Flk1-Cre*^*Breier*^*;tdTomato* mice showed that *Flk1-Cre*^*Breier*^ was specifically expressed in ECs with few, if any, signs of myeloid expression in the retina as well as in bone marrow, bone and skeletal muscle (Fig 4A and 4B). This remarkable endothelial specificity is likely due to the limited set of *Flk1* regulatory promoter regions used to generate the *Flk1-Cre*^*Breier*^ line, although *Flk1* expressing progenitor cells give rise to endothelial, hematopoietic and muscle lineages [31]. In *Flk1-Cre*^*Breier*^*;Lrp5*^*fl/-*^ CKO mice 43.8% of the retinal area exhibited similar vascular abnormalities as *Tie2-Cre;Lrp5*^*fl/fl*^ CKO mice, including absent intraretinal vessel layers, persistent hyaloid vessels and neovascularization in the NFL (Fig 4C and S4 Movie). In the retinal OPL of these CKO mice, areas with normal capillaries were often close to areas lacking any vascular development (Fig 4C). This suggests that horizontal endothelial growth in the OPL alone is insufficient to form and extend a large capillary network to areas where no vertical branches developed from the NFL. In addition, when one *tdTomato* allele was incorporated in these CKO mice, the vessels in areas with vascular abnormalities were always tdTomato^+^ while normal areas often included many tdTomato negative vessel branches (Fig 4D). This indicates that LRP5 functions autonomously in retinal ECs. Collectively, our data provide the first direct evidence that ECs are the primary cells that cause retinal hypovascularization during development and chaotic neovascularization in adulthood when LRP5 signaling is lost. In addition, based on the unique endothelial specificity of *Flk1-Cre*^*Breier*^, combined with other studies showing endothelial requirement for Norrin-FZD4-β-catenin signaling in retinal vascularization, our data further suggest that the Norrin-induced canonical Wnt pathway functions through endothelial LRP5.

### Conditional Restoration of LRP5 Signaling in Endothelial Cells Rescues the Vascular Defects in *Lrp5*^*-/-*^ Mice

**Fig 5:**
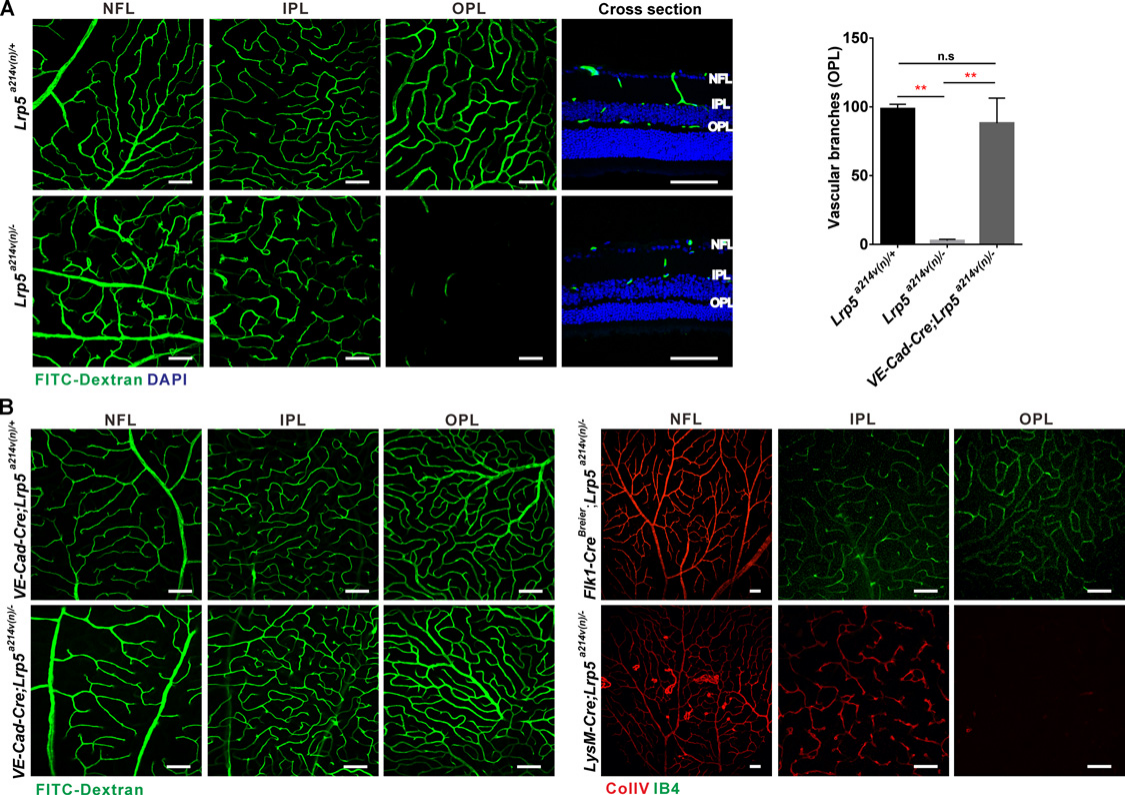
Conditionally restoring *Lrp5* in endothelial but not myeloid cells rescues retinal vascular defects in *Lrp5*^*-/-*^ mice. (A) FITC-Dextran perfusion showing distorted NFL and IPL vessels and lack of OPL vascular development in *Lrp5*^*a214v(n)/-*^ hypomorph mice (lower panels) compared to controls (upper panels) both in retinal whole mount (4w) and cross section (2m) images. (B) *VE-Cad-Cre;Lrp5*^*a214v(n)/+*^ (upper left panels, FITC-Dextran perfusion), *VE-Cad-Cre;Lrp5*^*a214v(n)/-*^ (lower left panels, FITC-Dextran perfusion) and *Flk1-Cre;Lrp5*^*a214v(n)/-*^ (upper right panels, red: ColIV, green: IB4) mice all developed a normalized three-tier retinal vascular structure, while *LysM-Cre;Lrp5*^*a214v(n)/-*^ (lower right panels, red: ColIV) mice displayed similar retinal vascular abnormalities compared to control *Lrp5*^*a214v(n)/-*^ mice (A, lower panels). Quantification of vascular branch points in OPL is shown in graph at right; **P<0.01, ns not significant. All mice are between 4 to 5 weeks of age except otherwise labeled. Scale bars = 100nm.

If loss of LRP5 in ECs alone is sufficient to cause the vascular defects observed in *Lrp5*^-/-^ mice, re-expressing LRP5 only in ECs in the *Lrp5*^-/-^ background should rescue the defective retinal vasculature in these mice. To test this hypothesis, we took advantage of an *Lrp5* knock-in hypomorph allele, *Lrp5* ^*a214v(n)*^, where *Lrp5* is transcribed at a very low level [21]. Following *Cre*-mediated removal of a neo cassette, this allele is converted into a high bone mass-causing allele (*Lrp5* ^*a214v*^*)* that has been shown to be comparable to WT LRP5 in its ability to transduce canonical Wnt signaling [32]. Retinal angiography with FITC-dextran perfusion showed that the *Lrp5* hypomorphic mice displayed retinal vascular defects similar to those of *Lrp5*^-/-^ mice. Adult *Lrp5*^a214v(neo)/-^ mice had retinas lacking OPL vascular development with their NFL and IPL vessels varying from normal to defective as in *Lrp5*^-/-^ mice, albeit to a lesser degree (Fig 5A and S5 Movie). Neovascularization in the NFL was also less prominent in *Lrp5*^*a214v(n)/-*^ than in *Lrp5*^-/-^ mice. Conditional restoration of LRP5 expression in ECs using *Flk1-Cre*^*Breier*^ or *VE-Cad-Cre* (Fig 5B and S6 Movie) in *Lrp5*^*a214v(n)/-*^ mice, restored normal retinal vascular development, while conditional restoration of *Lrp5* in myeloid cells with *LyzM-Cre* had no such effect (Fig 5B). These data strongly indicate that EC-derived LRP5 alone is sufficient for its control in normal retinal vascular development.

### Endothelial Cell-Derived LRP5 Does Not Share Redundant Roles with LRP6 in Its Regulation of Retinal Vascular Development

**Fig 6:**
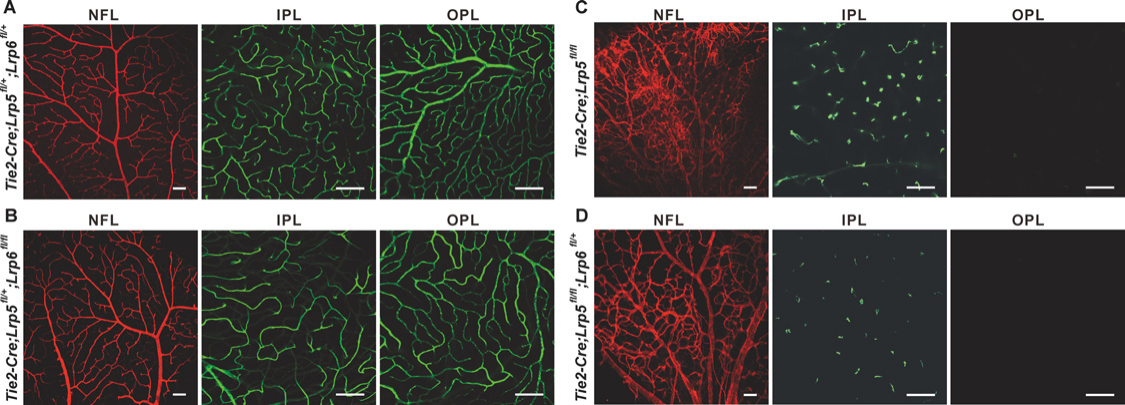
Endothelium-derived *Lrp6* is dispensable for retinal vascular development. Whole mount IF staining of ColIV (red) and FITC-Dextran perfusion showing (A) Retinal vasculature of *Tie2-Cre;Lrp5*^*fl/+*^*;Lrp6*^*fl/+*^ CKO mice. (B) Retinal vasculature of *Tie2-Cre;Lrp5*^*fl/+*^*;Lrp6*^*fl/fl*^ CKO mice. (C) Retinal vasculature of *Tie2-Cre;Lrp5*^*fl/fl*^ CKO mice. (D) Retinal vasculature of *Tie2-Cre;Lrp5*^*fl/fl*^*;Lrp6*^*fl/+*^ CKO mice. All mice are 8 weeks of age. Scale bars = 100nm.

LRP5 and LRP6 often serve as interchangeable co-receptors for the canonical Wnt pathway and share redundant roles in many developmental or pathological processes [22]. Activation of Norrin-FZD4-β-catenin signaling requires the presence of either LRP5 or LRP6 *in vitro* [14]. Endothelial deletion of *Lrp6* in the background of *Lrp5*^*-/-*^ mice resulted in severely attenuated brain vascularization and bleeding, but had little impact on the retinal vascular defects seen in *Lrp5*^*-/-*^ mice [17]. To further examine whether LRP5 and LRP6 have similar redundant functions in ECs during retinal vascular development, we deleted different copies of *Lrp5* and *Lrp6* with the most robust endothelial *Cre* line–*Tie2-Cre*–among the three that were used in this study. As shown in Fig 6A and 6B, when a single copy of *Lrp5* is deleted in ECs, additional removal of either one or both copies of *Lrp6* had no impact on retinal vascular development. Similarly, when both copies of *Lrp5* were deleted in ECs, adding a deletion of one *Lrp6* allele had no impact on the observed vascular defects in *Tie2-Cre;Lrp5*^*fl/fl*^ CKO mice (Fig 6C and 6D). Our data are consistent with the Zhou et al. study [17] and further suggest that while LRP5 is essential for retinal vascularization, LRP6 has a dispensable role in this process *in vivo*. This further indicates that the Norrin-FZD4-β-catenin pathway regulates retinal vascular development *in vivo* exclusively through LRP5 and not LRP6.

## Discussion

In the current study, we report that loss of LRP5 causes a profound change in the retinal vasculature including both hypovascularization and neovascularization. Through the use of multiple genetic models we demonstrate that specific endothelial deletion of *Lrp5* recapitulates the retinal vascular defects observed in *Lrp5*^-/-^ mice, while specific endothelial restoration of LRP5 in *Lrp5*^-/-^ mice rescues those defects. This suggests that LRP5 regulates an essential retinal vascularization program solely through ECs. In addition, LRP5 likely exerts a dosage dependent effect as the severity of the retinal vascular defects in *Lrp5* hypomorphic mice was between that of WT and *Lrp5*^-/-^ mice. Finally, LRP6 is completely dispensable in this process.

The overlapping ocular manifestations and genetic causes in OPPG, FEVR and ND patients strongly suggest a common genetic pathway involved in the pathogenesis of the disorders. Our findings that the retinal vascular defects in *Lrp5*^-/-^ mice also highly resemble those in *Ndp*^*-*^ and *Fzd4*^*-/-*^ mice [13,14,16] further confirm the critical regulation by each component of the ligand (Norrin)—receptor (FZD4)—co-receptor (LRP5) trio in the canonical Wnt pathway. Although Norrin-FZD4 has been shown to control retinal vascularization in ECs through β-catenin, how exactly LRP5 functions in this process remained to be determined. In addition, the use of *Tie2-Cre*, a *Cre* line that shows robust recombination in both endothelial and hematopoietic cells, in conditional knockout studies of *Fzd4* has left open the possibility of non-EC contributions to the essential regulation of Norrin-FZD4-mediated retinal vascular development. In the present study, we exclude any essential contribution of retinal myeloid/microglial cells to LRP5-mediated retinal vascularization. Using the highly endothelial specific *Flk1-Cre*^*Breier*^ line and *Flk1-Cre*^*Breier*^*;Lrp5*^*fl/-*^ and *Flk1-Cre*^*Breier*^*;Lrp5*^*a214v(n)/-*^ mice, we directly demonstrate an endothelial autonomous function of LRP5, as well as the Norrin-FZD4-LRP5-β-catenin pathway, during retinal vascular development.

Interestingly, although both LRP5 and LRP6 function as interchangeable essential co-receptors for activation of β-catenin signaling by Norrin-FZD4 *in vitro* [14] and have redundant functions in canonical Wnt signaling-mediated brain vascular formation *in vivo* [17], our study shows that LRP5, but not LRP6, is the critical co-receptor in Norrin-FZD4-β-catenin-mediated retinal vascularization, consistent with a previous report [17]. The differential requirement for LRP5 and LRP6 in the retina and brain could reflect different availability of the Wnt ligands in the two tissues during vascular development or different adaptation to additional regulation from other local factors, such as macrophages in the eye and oxygen levels in the brain.

The lack of a phenotype in *VE-Cad-Cre;Lrp5*^*fl/-*^ CKO mice is likely associated with incomplete Cre recombination and a wild type EC rescue effect. However, additional factors may also contribute, because in *Flk1-Cre*^*Breier*^*;Lrp5*^*fl/-*^ CKO mice, we often observed retinal areas with fully developed OPL vessels next to areas with no vessels. The fact that horizontal branching of capillaries alone is not sufficient to vascularize a large area in the OPL indicates that different cues may guide ECs to grow vertically into the OPL and horizontally throughout the OPL. Angiopoietin 2 (ANG2) has been shown to be essential for hyaloid vessel regression and OPL vascular development as *Ang2*^*-/-*^ mice lack OPL vasculature and have persistent hyaloid vessels [33,34], two major abnormalities observed in *Lrp5*^*-/-*^ retinas as well. This suggests that ANG2 may have an important interaction with Norrin-FZD4-LRP5 signaling in regulating retinal vascular development. Several lines of evidence support this enticing possibility. During hyaloid vessel regression, ANG2 critically stimulates WNT7B expression in macrophages, which is essential for inducing hyaloid EC death (also see the next paragraph); ANG2 is highly expressed in endothelial tip cells [35] and the leading edge of proliferating vessels [36]. Meanwhile, it is also significantly upregulated in retinal horizontal cells located at the outer border of the inner nuclear layer right at the time when OPL vessels start to develop [37]. Whether cooperation of ANG2 and WNT in macrophages also takes place in ECs during retinal endothelial outgrowth, with ANG2 either being a major endothelial target of Norrin-FZD4-LRP5 signaling or serving as a major guidance cue that requires the endothelial Norrin-FZD4-LRP5 pathway, remains an open question.

Defective regression of hyaloid vessels is a feature of *Ndp*^*-*^, *Fzd4*^*-/-*^ and *Lrp5*^*-/-*^ retinas [14,38,39]. This indicates that signaling from Norrin through FZD4-LRP5 is critical for regression of the hyaloid vasculature. Macrophage-derived WNT7B, also essential for apoptosis of hyaloid ECs, stimulates cell cycle entry through FZD4-LRP5, a crucial step in the subsequent ANG2-induced cell death [34]. Whether Norrin and WNT7B regulate hyaloid apoptosis through exactly the same canonical Wnt pathway remains to be determined. However, it is possible that the same Norrin-FZD4-LRP5 pathway regulates both retinal vascular outgrowth and hyaloid vessel cell death to ensure coordinated control of retinal vessels and appropriate local oxygen and VEGF levels. How could Norrin, a sticky matrix-associated protein primarily secreted by Muller glia cells, directly regulate apoptosis in hyaloid ECs? The possible answer to this question is provided by the findings that Norrin can be expressed by macrophages [40] and a large number of macrophages are directly in contact with hyaloid vessels during the regression phase. Therefore, it is enticing to hypothesize that macrophages are the major source of Norrin at hyaloid vessels. Perhaps the additional control through macrophage WNT7B ensures that macrophages are available for the aftermath of endothelial cell death, and does so through an efficient utilization of the same FZD4-LRP5 complex. Whether ANG2 also serves as a regulator of retinal EC growth and hyaloid EC apoptosis in coordination with Norrin-FZD4-LRP5 signaling awaits further investigation.

The rescue results using *Lrp5*^*a214v(n)*^ hypomorphic mice have important therapeutic implications for OPPG patients. Expression of a single *Lrp5* ^*a214v*^ allele in osteocytes is sufficient to cause high bone mass in mice [21], while expression of the same *Lrp5*^*a214v*^ allele in ECs of both *Lrp5* ^a214v/-^ and *Lrp5* ^a214v/+^ mice results in normalized retinal vascular development (Fig 5B). This suggests that the retinal vasculature is less sensitive to the effects of the *Lrp5* ^*a214v*^ allele compared to bone. Therefore, therapeutic strategies, based on manipulation of LRP5 or canonical Wnt signaling, may be effective in targeting both osteoporosis and retinal vascular defects in OPPG patients, with the retinal vasculature being more resistant to potential side effects.

With increasing prevalence of diabetes, DR has become a leading cause of blindness in adults in the western world. A key feature of DR is retinal vascular abnormalities including capillary leakage, microaneurysms, hemorrhages and fibrovascular tissue formation [37]. These vascular problems in turn lead to disruption or loss of vision. Interestingly, many of these neovascularization features are also presented in *Lrp5*^-/-^ mice. In addition, loss of LRP5, Norrin, or FZD4 causes decreased coverage of mural cells to retinal ECs [14,17], which is also considered a key attribute to the early phase of DR leading to loss of blood-retinal barrier and increased permeability [37]. What role does Wnt signaling play in developmental neovascularization caused by loss of gene function as in *Lrp5*^*-/-*^ retinas compared with pathological neovascularization as in DR? This study shows that loss of LRP5 leads to formation of retinal neovascular tufts and overgrowth during development. In contrast, pathological neovascularization in oxygen-induced retinopathy (OIR) mice is associated with upregulation of FZD4 and LRP5, and loss of *Lrp5* function reduces the neovasculature in the mice [40]. On the other hand, intravitreal injection of Norrin reduces neovascular tufts in the OIR model [41]. Further work is clearly needed to resolve these contradictory findings, but genetic models such as the mice used in this study offer tools for mechanistic investigations and therapeutic studies of retinal neovascularization that could benefit patients with DR.

## Conclusions

With genetic tools of great specificity, we demonstrate that mice lacking LRP5 only in endothelial cells recapitulate the retinal vascular defects seen in mice that have LRP5 totally knocked out. Additionally, when LRP5 function is reinstalled only in these endothelial cells in LRP5 knockout mice, the retinal vascular abnormalities are completely rescued. Thus, an endothelial autonomous function of LRP5 is a key component of the cellular mechanisms underlying OPPG and likely diabetic retinopathy as well.

## Materials and Methods

### Ethics Statement

All animal experiments were performed in compliance with NIH's Guide for the Care and Use of Laboratory Animals and Guidelines from the Harvard University Institutional Animal Care and Use Committee. The study was approved by the Harvard Medical School Institutional Animal Care and Use Committee (Protocol number 02074). Mice were deeply anesthetized with ketamine (150 mg/kg body weight) and xylazine (15mg/kg body weight). For euthanasia CO2 asphyxiation (longer exposure) was followed by decapitation.

### Animals

*Lrp5*^*-/-*^ [42] and *Lrp5*^*a214v(neo)/+*^ [21] mice were generated as described. *Lrp5*^*fl/fl*^ and *Lrp6*^*fl/fl*^ [22], *Flk1*^*Breier*^*-Cre* [30], *Rx-Cre* [23] and *CD11b-Cre* [29] mice were kindly provided by Drs. Bart O. William, Kevin P. Campbell, Eric Swindell and Roland Baron, respectively. *VE-Cad-Cre* [24], *Tie2-Cre* [25], *LysM-Cre* [28] and tdTomato [27] mice were purchased from the Jackson Laboratory. *Flk1*^*Breier*^*-Cre*, *Rx-Cre*, *CD11b-Cre*, *VE-Cad-Cre* and *Tie2-Cre* mice were all transgenic lines generated by fusing the *Cre* gene to a fragment of the promoter sequence of *Flk1*, *Rx*, *CD11b*, *VE-Cad* and *Tie2*, respectively. *LysM-Cre* mice were knock-ins, generated by targeted insertion of the *Cre* cDNA into the endogenous M lysozyme locus. When animals from different genetic backgrounds were crossed, littermate controls were used to avoid confounding effects.

### Antibodies

The following antibodies were used for immuno-staining: ColIV (EMD Millipore), CD31 (BD Pharmingen), fibronectin (Sigma), F4/80 (Invitrogen), Alexa Fluor 488 (or 555) conjugated anti-rabbit secondary antibody (Invitrogen).

### Immunofluorescence Microscopy

For whole-mount retina IF staining, eyes were fixed in 4% PFA in PBS for 30min at room temperature (RT) or overnight at 4°C. Retinas were dissected, fixed for an additional 30min at RT, permeabilized with PBS containing 1% Triton-100 for 1 hr at RT, blocked for 1–4 hrs in PBST (0.2% Triton-100 in PBS) containing 2% BSA at RT and incubated with primary antibodies or IB4-biotin (Sigma) overnight at 4°C in PBST containing 1% BSA. After thorough washes in PBST, retinas were incubated with fluorescent secondary antibodies or streptavidin-Alexa Fluor dye conjugates together with Hoechst 33342 (Molecular Probes) overnight at 4°C, washed with PBST and flat mounted with mounting medium (Vector Lab). Images were acquired with either MetaMorph software on a Nikon 80i microscope or Nikon Elements software on a Nikon A1R laser scanning confocal microscope. Z stack images were acquired with a step size of 0.5 microns using a Nikon Ti-E motorized A1R microscope with Perfect Focus System. Z series were reconstructed as a video with Nikon Elements.

For quantification of vascular sprouting in the retina of 5-day old mice, the numbers of vascular sprouts at the edge of the growing vascularized area were counted in 20X-magnified images of IB4-biotin-stained whole mounts from 6 control and 6 *Lrp5*^*-/-*^ mice as described [40]. For quantification of vascular density within the OPL, the number of vascular branch-points were counted in 20X-magnified images of FITC-Dextran-perfused retinas from four *Lrp5*^*fl/fl*^, *VE-Cad-Cre;Lrp5*^*fl/-*^, *Lrp5* ^*a214v(n)/+*^, *Lrp5* ^*a214v(n)/-*^, *and VE-Cad-Cre;Lrp5* ^*a214v(n)/-*^ mice.

### FITC-Dextran Perfusion

2 ml of PBS containing 500 units of heparin were perfused through the heart of a deeply anesthetized mouse, followed by 1 ml PBS containing 15mg/ml FITC conjugated Dextran (Sigma) and 4% paraformaldehyde (PFA), prepared immediately prior to use. Eyes were post-fixed in 4% PFA in PBS overnight at 4°C and retinas were dissected, post-fixed in 4% PFA in PBS for 30min at RT and flat-mounted in Mounting medium (Vector Lab).

### Electron Microscopy

Mice were anesthetized and perfused through the heart with a solution containing 2% PFA, 2.5% glutaraldehyde and 0.1M sodium cacodylate (pH 7.4). Eyes were enucleated and fixed in the same solution overnight at 4°C. Retinas were dissected out, post-fixed for 2hrs at RT, cut in half across the optical nerve center, treated with 1% osmiumtetroxide and 1% potassium ferrocyanide, en bloc stained with 1% uranyl acetate, dehydrated and embedded in Epon (Marivac Inc., Canada). 80nm thin sections were cut and stained with uranylacetate and lead citrate. The sections were analyzed and micrographs were acquired with a JEOL 1200EX electron microscope.

### ELISA

Dissected retinas were rinsed with ice-cold PBS and residual PBS was removed by quickly tapping the retina to the dry area of a petri dish several times. Retinas were immediately homogenized in ice-cold M-PER lysis buffer (Thermo Scientific) containing proteinase and phosphatase inhibitors (Thermo Scientific) and stored at -80°C. After a freeze-thaw cycle, homogenates were centrifuged and the supernatants were used to quantify VEGF levels using the mouse VEGF Quantikine ELISA kit (R&D Systems). VEGF values were normalized to the total retinal protein amount analyzed by Pierce Coomassie Protein Assay Kit (Thermo Scientific). All samples were measured in duplicates.

### Statistical Analysis

Data between 2 groups were compared by unpaired two-tailed Student’s *t*-test. For the retinal VEGF protein levels, 2-way ANOVA with multiple comparisons was used. *P* values less than 0.05 were considered significant.

## Supporting Information

S1 Movie Retinal vasculature of *Lrp5*^*-/-*^ mice. FITC-Dextran perfusion.
(AVI)

S2 Movie Retinal vasculature of control mice. FITC-Dextran perfusion.
(AVI)

S3 Movie Retinal vasculature of *Rx-Cre;Lrp5*^*fl/-*^ CKO mice. FITC-Dextran perfusion.
(AVI)

S4 Movie Retinal vasculature of *Flk1-Cre;Lrp5*^*fl/-*^ CKO mice. IB4 whole mount IF staining.
(AVI)

S5 Movie Retinal vasculature of *Lrp5*^*a214v(n)/-*^ mice. FITC-Dextran perfusion.
(AVI)

S6 Movie Retinal vasculature of *VE-Cad-Cre;Lrp5*^*a214v(n)/-*^ mice. FITC-Dextran perfusion.
(AVI)

## References

[1] Stevens GA, White RA, Flaxman SR, Price H, Jonas JB, Keeffe J, et al Global prevalence of vision impairment and blindness: magnitude and temporal trends, 1990–2010. Ophthalmology. 2013;120: 2377–2384. 10.1016/j.ophtha.2013.05.025 23850093

[2] Gariano RF, Gardner TW. Retinal angiogenesis in development and disease. Nature. 2004;438: 960–966. 10.1038/nature04482 16355161

[3] Gilbert C. Retinopathy of prematurity: a global perspective of the epidemics, population of babies at risk and implications for control. Early Hum Dev. 2008;84: 77–82. 10.1016/j.earlhumdev.2007.11.009 18234457

[4] Grammas P, Riden M. Retinal endothelial cells are more susceptible to oxidative stress and increased permeability than brain-derived endothelial cells. Microvasc Res. 2003;65: 18–23. 12535867 10.1016/s0026-2862(02)00016-x

[5] Frontali M, Stomeo C, Dallapiccola B, Opitz JM, Reynolds JF. Osteoporosis‐pseudoglioma syndrome: Report of three affected sibs and an overview. Am J Med Genet. 1985;22: 35–47. 3931475 10.1002/ajmg.1320220104

[6] Gong Y, Vikkula M, Boon L, Liu J, Beighton P, Ramesar R, et al Osteoporosis-pseudoglioma syndrome, a disorder affecting skeletal strength and vision, is assigned to chromosome region 11q12-13. Am J Hum Genet. 1996;59: 146–151. 8659519 PMC1915094

[7] Gong Y, Slee RB, Fukai N, Rawadi G, Roman-Roman S, Reginato AM, et al LDL receptor-related protein 5 (LRP5) affects bone accrual and eye development. Cell. 2001;107: 513–523. 11719191 10.1016/s0092-8674(01)00571-2

[8] Nikopoulos K, Venselaar H, Collin RW, Riveiro‐Alvarez R, Boonstra FN, Hooymans JM, et al Overview of the mutation spectrum in familial exudative vitreoretinopathy and Norrie disease with identification of 21 novel variants in FZD4, LRP5, and NDP. Hum Mutat. 2010;31: 656–666. 10.1002/humu.21250 20340138

[9] Chen ZY, Battinelli EM, Fielder A, Bundey S, Sims K, Breakefield XO, et al A mutation in the Norrie disease gene (NDP) associated with X-linked familial exudative vitreoretinopathy. Nat Genet. 1993;5: 180–183. 8252044 10.1038/ng1093-180

[10] Robitaille J, MacDonald ML, Kaykas A, Sheldahl LC, Zeisler J, Dubé M, et al Mutant frizzled-4 disrupts retinal angiogenesis in familial exudative vitreoretinopathy. Nat Genet. 2002;32: 326–330. 12172548 10.1038/ng957

[11] Toomes C, Bottomley HM, Jackson RM, Towns KV, Scott S, Mackey DA, et al Mutations in LRP5 or FZD4 underlie the common familial exudative vitreoretinopathy locus on chromosome 11q. 2004;74: 721–730. 15024691 10.1086/383202 PMC1181948

[12] Warburg M. Norrie's disease. A congenital progressive oculo-acoustico-cerebral degeneration. Acta Ophthalmol (Copenh). 1966: Suppl 89:1–47. 6013082

[13] Xu Q, Wang Y, Dabdoub A, Smallwood PM, Williams J, Woods C, et al Vascular development in the retina and inner ear: control by Norrin and Frizzled-4, a high-affinity ligand-receptor pair. Cell. 2004;116: 883–895. 15035989 10.1016/s0092-8674(04)00216-8

[14] Ye X, Wang Y, Cahill H, Yu M, Badea TC, Smallwood PM, et al Norrin, frizzled-4, and Lrp5 signaling in endothelial cells controls a genetic program for retinal vascularization. Cell. 2009;139: 285–298. 10.1016/j.cell.2009.07.047 19837032 PMC2779707

[15] Wang Y, Rattner A, Zhou Y, Williams J, Smallwood PM, Nathans J. Norrin/Frizzled4 signaling in retinal vascular development and blood brain barrier plasticity. Cell. 2012;151: 1332–1344. 10.1016/j.cell.2012.10.042 23217714 PMC3535266

[16] Junge HJ, Yang S, Burton JB, Paes K, Shu X, French DM, et al TSPAN12 regulates retinal vascular development by promoting Norrin-but not Wnt-induced FZD4/β-catenin signaling. Cell. 2009;139: 299–311. 10.1016/j.cell.2009.07.048 19837033

[17] Zhou Y, Wang Y, Tischfield M, Williams J, Smallwood PM, Rattner A, et al Canonical WNT signaling components in vascular development and barrier formation. J Clin Invest. 2014;124: 3825–3846. 10.1172/JCI76431 25083995 PMC4151216

[18] Tang Y, Harrington A, Yang X, Friesel RE, Liaw L. The contribution of the Tie2 lineage to primitive and definitive hematopoietic cells. Genesis. 2010;48: 563–567. 10.1002/dvg.20654 20645309 PMC2944906

[19] Xia C, Yablonka-Reuveni Z, Gong X. LRP5 is required for vascular development in deeper layers of the retina. 2010;5: e11676 10.1371/journal.pone.0011676 20652025 PMC2907392

[20] Roberts WG, Palade GE. Increased microvascular permeability and endothelial fenestration induced by vascular endothelial growth factor. J Cell Sci. 1995;108 (Pt 6): 2369–2379. 7673356 10.1242/jcs.108.6.2369

[21] Cui Y, Niziolek PJ, MacDonald BT, Zylstra CR, Alenina N, Robinson DR, et al Lrp5 functions in bone to regulate bone mass. Nat Med. 2011;17: 684–691. 10.1038/nm.2388 21602802 PMC3113461

[22] Zhong Z, Baker JJ, Zylstra‐Diegel CR, Williams BO. Lrp5 and Lrp6 play compensatory roles in mouse intestinal development. J Cell Biochem. 2012;113: 31–38. 10.1002/jcb.23324 21866564 PMC3245350

[23] Swindell EC, Bailey TJ, Loosli F, Liu C, Amaya‐Manzanares F, Mahon KA, et al Rx‐Cre, a tool for inactivation of gene expression in the developing retina. Genesis. 2006;44: 361–363. 16850473 10.1002/dvg.20225

[24] Alva JA, Zovein AC, Monvoisin A, Murphy T, Salazar A, Harvey NL, et al VE‐Cadherin‐Cre‐recombinase transgenic mouse: A tool for lineage analysis and gene deletion in endothelial cells. 2006;235: 759–767. 16450386 10.1002/dvdy.20643

[25] Kisanuki YY, Hammer RE, Miyazaki J, Williams SC, Richardson JA, Yanagisawa M. Tie2-Cre transgenic mice: a new model for endothelial cell-lineage analysis in vivo. Dev Biol. 2001;230: 230–242. 11161575 10.1006/dbio.2000.0106

[26] Zovein AC, Hofmann JJ, Lynch M, French WJ, Turlo KA, Yang Y, et al Fate tracing reveals the endothelial origin of hematopoietic stem cells. 2008;3: 625–636. 10.1016/j.stem.2008.09.018 PMC2631552 19041779

[27] Madisen L, Zwingman TA, Sunkin SM, Oh SW, Zariwala HA, Gu H, et al A robust and high-throughput Cre reporting and characterization system for the whole mouse brain. Nat Neurosci. 2010;13: 133–140. 10.1038/nn.2467 20023653 PMC2840225

[28] Clausen B, Burkhardt C, Reith W, Renkawitz R, Förster I. Conditional gene targeting in macrophages and granulocytes using LysMcre mice. Transgenic Res. 1999;8: 265–277. 10621974 10.1023/a:1008942828960

[29] Ferron M, Vacher J. Targeted expression of Cre recombinase in macrophages and osteoclasts in transgenic mice. Genesis. 2005;41: 138–145. 15754380 10.1002/gene.20108

[30] Licht AH, Raab S, Hofmann U, Breier G. Endothelium-specific Cre recombinase activity in flk-1-Cre transgenic mice. Dev Dyn. 2004;229: 312–318. 14745955 10.1002/dvdy.10416

[31] Motoike T, Markham DW, Rossant J, Sato TN. Evidence for novel fate of Flk1 progenitor: contribution to muscle lineage. Genesis. 2003;35: 153–159. 12640619 10.1002/gene.10175

[32] Ai M, Holmen SL, Van Hul W, Williams BO, Warman ML. Reduced affinity to and inhibition by DKK1 form a common mechanism by which high bone mass-associated missense mutations in LRP5 affect canonical Wnt signaling. Mol Cell Biol. 2005;25: 4946–4955. 15923613 10.1128/MCB.25.12.4946-4955.2005 PMC1140571

[33] Hackett SF, Wiegand S, Yancopoulos G, Campochiaro PA. Angiopoietin‐2 plays an important role in retinal angiogenesis. J Cell Physiol. 2002;192: 182–187. 12115724 10.1002/jcp.10128

[34] Rao S, Lobov IB, Vallance JE, Tsujikawa K, Shiojima I, Akunuru S, et al Obligatory participation of macrophages in an angiopoietin 2-mediated cell death switch. Development. 2007;134: 4449–4458. 18039971 10.1242/dev.012187 PMC3675770

[35] Felcht M, Luck R, Schering A, Seidel P, Srivastava K, Hu J, et al Angiopoietin-2 differentially regulates angiogenesis through TIE2 and integrin signaling. J Clin Invest. 2012;122: 1991–2005. 10.1172/JCI58832 22585576 PMC3366398

[36] Maisonpierre PC, Suri C, Jones PF, Bartunkova S, Wiegand SJ, Radziejewski C, et al Angiopoietin-2, a natural antagonist for Tie2 that disrupts in vivo angiogenesis. Science. 1997;277: 55–60. 9204896 10.1126/science.277.5322.55

[37] Armulik A, Genové G, Betsholtz C. Pericytes: developmental, physiological, and pathological perspectives, problems, and promises. 2011;21: 193–215. 10.1016/j.devcel.2011.07.001 21839917

[38] Ohlmann AV, Adamek E, Ohlmann A, Lütjen-Drecoll E. Norrie gene product is necessary for regression of hyaloid vessels. Invest Ophthalmol Vis Sci. 2004;45: 2384–2390. 15223821 10.1167/iovs.03-1214

[39] Kato M, Patel MS, Levasseur R, Lobov I, Chang BH, Glass DA II, et al Cbfa1-independent decrease in osteoblast proliferation, osteopenia, and persistent embryonic eye vascularization in mice deficient in Lrp5, a Wnt coreceptor. J Cell Biol. 2002;157: 303–314. 11956231 10.1083/jcb.200201089 PMC2199263

[40] Chen J, Stahl A, Krah NM, Seaward MR, Dennison RJ, Sapieha P, et al Wnt signaling mediates pathological vascular growth in proliferative retinopathy. Circulation. 2011;124: 1871–1881. 10.1161/CIRCULATIONAHA.111.040337 21969016 PMC3326389

[41] Tokunaga CC, Chen Y, Dailey W, Cheng M, Drenser KA. Retinal Vascular Rescue of Oxygen-Induced Retinopathy in Mice by NorrinRetinal Vascular Rescue of OIR in Mice by Norrin. Invest Ophthalmol Vis Sci. 2013;54: 222–229. 10.1167/iovs.12-10127 23188723

[42] Clement-Lacroix P, Ai M, Morvan F, Roman-Roman S, Vayssiere B, Belleville C, et al Lrp5-independent activation of Wnt signaling by lithium chloride increases bone formation and bone mass in mice. Proc Natl Acad Sci U S A. 2005;102: 17406–17411. 16293698 10.1073/pnas.0505259102 PMC1297659

